# Preference-based utility weights for the Individualized Neuromuscular Quality of Life Questionnaire (INQoL), with a focus on non-dystrophic myotonia (NDM)

**DOI:** 10.1007/s10198-024-01674-2

**Published:** 2024-02-28

**Authors:** Andrew Lloyd, Kim Rand, Cleo Pike, Crispin Ellis

**Affiliations:** 1 Acaster Lloyd Consulting Ltd, London, UK; 2 Health Services Research Unit, Akershus University Hospital, Lørenskog, Norway; 3 Maths in Health B.V., Klimmen, The Netherlands; 4 Lupin Healthcare (UK) Ltd, Slough, Berkshire UK

**Keywords:** Non-dystrophic myotonia, Quality of Life, Utility, Individualized Neuromuscular Quality of Life Questionnaire, Health, Pharmacoeconomics, I10

## Abstract

**Introduction:**

The Individualized Neuromuscular Quality of Life Questionnaire (INQoL) is used to measure quality of life in neuromuscular disorders such as non-dystrophic myotonia (NDM). Here we report methods to estimate utilities, with a focus on NDM, from this questionnaire based on two preference elicitation exercises.

**Methods:**

Eight items from the INQoL were selected with input from three neuromuscular disorder clinical experts with expertise in treating NDM. A discrete choice experiment (DCE) survey of UK general public respondents (*n* = 508) described outcomes defined by the INQoL items. The same 8 items were also valued using time trade-off (TTO) face-to-face interviews (*n* = 200). A hybrid regression modelling approach combined both datasets to inform the utility weights.

**Results:**

Hybrid modelling of DCE and TTO data in conjunction improved out-of-sample predictive accuracy. The selected INQoL utility model indicates substantial disutility associated with all eight dimensions of health, with the greatest losses associated with subjective items such as pain and depression.

**Discussion:**

The hybrid modelling approach allows us to combine data from the two methodologies and maximize the information from each to inform the utility weights for the INQoL. The TTO is the more conventional valuation method, but combined with the larger DCE study produced better descriptive coverage. This is a relatively novel method for estimating weights which we think is particularly well suited to economic evaluations of orphan drugs.

**Supplementary Information:**

The online version contains supplementary material available at 10.1007/s10198-024-01674-2.

## Introduction

In many countries, preference-based measures of health are used in economic evaluation to inform health policy [1, 2]. Preference-based measures are typically used to generate a value for health-related quality of life (HRQoL) that can be used to generate a quality adjusted life year (QALY). A QALY combines the value of quality of life with the length of life in each health state into a single index number, and can be used to capture changes in both mortality and morbidity of patients. The QALY provides a measure of the benefit of an intervention which can be compared to its incremental cost. Interventions can then be compared in terms of their incremental cost per QALY ratio.

The EQ-5D is the most widely used instrument for measuring HRQoL for QALY estimation worldwide [3], but it is a generic measure of HRQoL which may not reflect all the different ways that patients are affected. A condition-specific preference-based measure, as an alternative technique to measuring and valuing HRQoL, can be recommended when generic preference-based measures such as EQ-5D is either appropriate but data not available or is not appropriate in clinical use [1, 2, 4].

The Individualized Neuromuscular Quality of Life Questionnaire (INQoL) is a measure of HRQoL designed specifically for people with neuromuscular disease [5, 6]. The scale is used for people to rate the severity of symptoms and functional problems that they experience. Subsequent questions then explore the extent to which these issues affect or limit a patient’s day to day life. The INQoL is the only validated HRQoL questionnaire that refers specifically to the presence and impact of myotonic symptoms and has been repeatedly used in trials to assess the quality of life of people with non-dystrophic myotonia (NDM) [7–9]. However, the INQoL measure in its current form is not suitable for use in the estimation of QALYs because the scale is not scored as a preference weighted single index [10]. In rare diseases, such as in NDM, it is very difficult to collect additional HRQoL data outside of clinical trials to inform economic models. This is due to recruitment challenges, low disease awareness, and lack of funding for additional data collection, and therefore, it is very important to find solutions for using available trial data. In addition, the availability of data from a measure like INQoL which has been completed by patients themselves provides a solid basis for estimating utilities because it reflects patient experience. Neither HRQoL data from trials nor the use in clinical practice of EQ-5D in NDM has been found in the literature. This study, therefore, was designed to determine utility-based scoring weights using INQoL items which can be used for estimating QALYs, with a focus on NDM.

## Methods

### The INQoL questionnaire and sub-setting for preference elicitation

The INQoL questionnaire comprises 45 questions on 7 or 8-point scales, grouped in categories covering aspects of muscle weakness, muscle locking, pain, tiredness (fatigue), activities, independence, relationships, “how you feel” (anxiety, depression, frustration, self-esteem), “the way you look”, and aspects of treatment. In addition, the muscle weakness, locking, pain, and tiredness categories are preceded by yes/no questions as to whether any such symptoms are present, and a few questions in the treatment category have additional options, such as “I am not yet receiving treatment” and “I’m unsure”. Given the large number of questions and options within each, the descriptive system covers approximately 1.6 × 10^34^ unique responses, which poses a substantial challenge for use in preference elicitation. Such a lengthy instrument needs to be simplified to estimate utilities.

A review of the INQoL was completed to identify candidate items to include for valuation. The proposed item selection was discussed in interviews with three leading neuromuscular disorder clinical experts with expertise in treating NDM, who reviewed the process of selecting items and response levels, while an expert in utilities reviewed the proposed approach and provided feedback [11]. Consequently, items were selected from the INQoL which most closely aligned with EQ-5D in terms of its descriptive system in order to reflect the generic elements of health (e.g. physical function, pain, mental health, daily activities). The EQ-5D is very widely used to estimate utilities in cost-effectiveness analyses [3]. Eight items were selected to match the EQ-5D: Weakness (WE), Locking (LO), Daily activities (DA), Leisure (LE), Pain (PA), Fatigue (FA), Anxiety (AN), and Depression (DE). The clinical experts suggested that Fatigue (FA) should be included as it is one of the most frequently cited problems experienced by people with chronic illness [12–15]. The INQoL dimensions muscle weakness (WE) and muscle locking (LO) were included in the mapping to reflect mobility in the EQ-5D. In addition, locking was included because it was a key feature of NMD. Research studies have described the motor problems that NDM patients experienced, including significant difficulties in standing up quickly, running, and climbing stairs [15, 16]. Table 1 describes how the eight included INQoL dimensions line up with the dimensions of the EQ-5D. Further review of the selection of the items was carried out by a Delphi Panel exercise [17]. In this exercise nine clinical experts in treating NDM took part in a two-round Delphi panel. The results suggested that it is feasible to map domains of INQoL to EQ-5D, and confirmed the selection of the INQoL items.

**Table 1:** The INQoL items chosen to match the EQ-5D items

EQ-5D	INQoL
Mobility	How much weakness would you say you have in the muscles affected by your condition? How much muscle locking would you say you have at the moment?
Self-care (washing and dressing)	At the moment does your muscle condition affect your ability to do daily activities, e.g. washing, dressing, and housework?
Usual activities (leisure, work, social activities)	At the moment does your muscle condition affect your ability to do leisure activities?
Pain/discomfort	How much pain would you say you have at the moment? How much tiredness/ fatigue would you say you have at the moment?
Anxiety/depression	At the moment does your muscle condition make you feel anxious/ worried? At the moment does your muscle condition make you feel depressed?

Response options for items varied. Notably, the question scales for WE, LO, PA, and TI were preceded by a question as to the presence of any symptoms, and the 7-point scales started with levels “very little” and “some”; while DA, LE, AN, and DE were not preceded with any questions, and the subscales started with “Not at all” and “Slightly”. Appendix Figs. 1 and 2 show how the questions for muscle weakness (WE) and daily activities (DA) were presented. Table 2 describes the level structure for all eight included dimensions, and how they have been lined up in the statistical modelling.

**Table 2:**
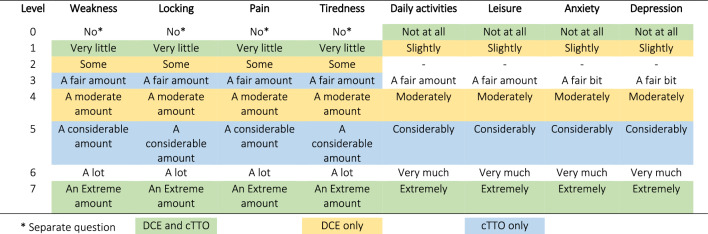
INQoL levels for the 8 dimensions used in the cTTO and DCE surveys

### General population preference elicitation: cTTO and DCE

Inspired by valuation studies for the EQ-5D-5L, general population preferences were elicited using two valuation methods: the composite time trade-off (cTTO) [18] and a discrete choice experiment (DCE). cTTO was administered face-to-face to 200 adult respondents from the general population, and DCE was administered online to 508 respondents in a survey hosted by Global Perspectives. Face-to-face TTO administration is costly, limiting the achievable sample size. In line with recommendations for TTO sample sizes garnered from valuation of EQ-5D health states suggesting that 100 observations per valued health state allows for an expected standard error of around 0.01 around mild to moderate health states, the study was designed to provide approximately 100 observations per valued state with two blocks of states for valuation [19]. Online DCE is considerably less costly, allowing for larger sample sizes and greater representativeness. Quota sampling was used to balance geographic distribution, gender, and ethnicity. Given the number of possible combinations allowed, even with the number of dimensions reduced to eight, covering all levels for all dimensions would reduce statistical power substantially, and was not considered feasible. The state selection was restricted to four levels, with intermediate levels interpolated, an approach supported by our methodology advisor.

In the DCE study 508 respondents were each administered 16 paired comparisons. The eight selected INQoL questions were combined with response choices using a published orthogonal design (http://neilsloane.com/oadir/). The orthogonal design required 32 choice questions using combinations of levels 1, 2, 4, and 7 for dimensions WE, LO, PA, and TI, and 0, 1, 4, and 7 for dimensions DA, LE, AN, and DE. The second choice in each question was determined by shifting the first choice (so level 1 in choice A becomes level 2 in choice B). This is a simple method for producing efficient pairs of choices in main-effects models described by Street and colleagues [20]. The order of questions was randomized and half of the participants completed questions 1–16 and the remaining half 17–32. An example of a DCE choice set is found in supplementary Fig. 1.

In the cTTO study, each respondent was administered 16 INQoL states for valuation, organized in two blocks, for a total of 32 unique states, selected using the same orthogonal design as in the DCE survey. The states were designed using combinations of levels 1, 3, 5, and 7 for dimensions WE, LO, PA, and TI, and 0, 3, 5, and 7 from dimensions DA, LE, AN, and DE. These levels were selected partly in order to get more level coverage. Trained field-based interviewers (all with prior TTO interviewer experience) recruited a convenience sample of the UK general public to participate in TTO interviews to assess each state. Participant recruitment was monitored to ensure that the sample reflected the UK general population. Written informed consent was taken. The participants completed some background questions. The TTO task was explained in detail to participants and the interview consisted of completing assessments of each health state. An example of a TTO vignette is found in supplementary Fig. 2.

### Statistical modelling

The two datasets (DCE and cTTO) were amenable to modelling both separately and combined in a cTTO + DCE hybrid model [21]. In either case, some interpolation would be required, covering the dimension/level combinations not present in the analyses. Given the large number of dimension/level combinations present, a complete main-effects model would require 28 primary coefficients for each type of data modelled alone, and 44 in a cTTO + DCE hybrid model, rendering the models highly susceptible to overfitting. Consequently, we tested model candidates with different levels of constraints and used cross-validation for model selection, in an extension of procedures used previously in EQ-5D-5L valuation studies [22].

We tested four primary model candidates, applied separately to cTTO and DCE, and used in cTTO + DCE hybrid combinations, all of which were tested with and without intercepts:

#### Main-effects models

Main-effects models employ one parameter for each observed imperfect level/dimension combination, yielding 3*8 + 4 = 28(+ intercept) parameters for cTTO and DCE alone, on the form

$$\alpha + \sum\limits_{{l,{\text{dim}}}} {\left( {{\text{dim}}_{l} \cdot \beta _{{{\text{dim}},l}} } \right)} + \varepsilon .$$

This model format is the not only most flexible, but also most susceptible to overfitting and non-monotonicity. Furthermore, with the different levels used in DCE and cTTO, the total number of primary parameters increases to 44.

To avoid non-monotonicity, the primary parameters in this format were fitted using box constraints, allowing them to vary in the interval [0, ∞] (positive, as the models were fitted using disutilities).

#### Linear models

The most constrained models tested, used a single parameter per dimension, assuming linearity in levels. If dim denotes observed variables for each dimension (integers from 0 to 7), they take the form

$$\alpha +\frac{{\sum }_{{\text{dim}}} \left({\text{dim}}\cdot {\beta }_{\mathrm{ dim}}\right)}{7}+\varepsilon .$$

This yields a minimum of 8 parameters (+ intercepts). The division by 7 is included for easier comparison with CALE model coefficients, as the dimension coefficients in CALE take the disutility of the highest level, i.e. level 7.

#### Cross-attribute level-effect (CALE) models

CALE models are constrained main effect models, in which coefficients for dimensions are multiplied by coefficients specific to levels, which apply across dimensions.

The general format is

$$\alpha + \sum\limits_{l} {\left( {\beta _{l} \sum\limits_{{{\text{dim}}}} {{\text{dim}}_{l} } {\mkern 1mu} \beta _{{{\text{dim}}}} } \right)} + \varepsilon .$$

Two CALE models were tested: one in which level coefficients were shared across all dimensions (referred to as CALE) and one in which level parameters were held separate for the two sets of dimensions with different presentational formats (WE, LO, PA, TI and DA, LE, AN, DE), referred to as CALEsep.

#### DCE models

DCE models were fitted using conditional logistic regression. Let x denote the independent variables of the model, β the modelled coefficients, and xβ + ε represent the regression model. As cTTO and DCE coefficients given the same model setup could have different scales, let β’ be the coefficients for DCE. Furthermore, let j denote observations and jЄD represent the dichotomous observations (i.e. DCE observations). The log-likelihood function maximized was

$$\underset{\beta \mathrm{^{\prime}}}{{\text{argmax}}}(D)=\sum_{j\mathrm{\epsilon D}}\left\{{\text{ln}}\left(\frac{1}{1+{e}^{-x\beta \mathrm{^{\prime}}}}\right){y}_{j}+{\text{ln}}\left(\frac{{e}^{-x\beta \mathrm{^{\prime}}}}{1+{e}^{-x\beta \mathrm{^{\prime}}}}\right)\right\}.$$

#### cTTO models

The cTTO design employed allows a lowest possible expressed value of – 1. However, some respondents would likely assign lower values if given the option. Values at – 1 were therefore considered left censored (i.e. in the interval [– ∞, – 1]). As health preference data are conventionally modelled using disutilities, i.e. *u*^*ʹ*^ = – (*u* – 1), values were handled as right censored at 2 (i.e. in the interval [2, ∞]). If *jЄC* represents discrete continuous observations, and *jЄR* represents right-censored observations, the log-likelihood function that was maximized was

$$\underset{\beta }{{\text{argmax}}}(C,R)=-\frac{1}{2}\sum_{j\mathrm{\epsilon C}}\left\{{\text{ln}}\left(2\pi {\sigma }_{j}^{2}\right)+{\left(\frac{{y}_{j}-x\beta }{{\sigma }_{j}}\right)}^{2}\right\}+ \sum_{j\mathrm{\epsilon R}}ln\left\{\phi \left(\frac{-\left({y}_{Rj}-x\beta \right)}{{\sigma }_{j}}\right)\right\} .$$

#### Hybrid models

The hybrid model maximizes the product likelihood (sum log-likelihood) over DCE and TTO data, given a set of shared parameters and a linear transform between the two, using the parameter *θ*.

$$\underset{\beta }{{\text{argmax}}}=-\frac{1}{2}\sum_{j\mathrm{\epsilon C}}\left\{{\text{ln}}\left(2\pi {\sigma }_{j}^{2}\right)+{\left(\frac{{y}_{j}-x\beta }{{\sigma }_{j}}\right)}^{2}\right\}+ \sum_{j\mathrm{\epsilon R}}ln\left\{\phi \left(\frac{-\left({y}_{Rj}-x\beta \right)}{{\sigma }_{j}}\right)\right\} +\sum_{j\mathrm{\epsilon D}}\left\{{\text{ln}}\left(\frac{1}{1+{e}^{\left(-x\beta /\theta \right)}}\right){y}_{j}+{\text{ln}}\left(\frac{{e}^{\left(-x\beta /\theta \right)}}{1+{e}^{\left(-x\beta /\theta \right)}}\right)\right\}.$$

#### Heteroscedasticity in TTO data

To account for heteroscedasticity in modelling cTTO data, the error term used in the normal distribution-based likelihood function was modified to allow variation linear in mean disutility. If *x*_*βj*_ is the predicted value for observation *j*, this was done by applying the following formula to the standard deviation in the normal distribution-based likelihood estimation:

$${\sigma }_{j}={\alpha }_{\sigma }+{\beta }_{\sigma }\times {X}_{\beta j.}$$

### Model selection

To compare model performance and limit the risk of overfitting, model selection was done by means of out-of-sample predictive accuracy, estimated using cross-validation at the level of individual health states (cTTO) or health state pairs (DCE).

For cTTO, the steps were as follows:

1. Exclude all observations of one of the 32 employed health states.
2. Fit all candidate models to the remaining 31 health states (and DCE data, in the case of hybrid models).
3. Predict values for the excluded health state.
4. Compare the predicted values to the censored mean value for the excluded health state in terms of log-likelihood, root-mean square error (RMSE), mean absolute error (MAE), Pearson’s product moment correlation (R), Fisher’s intra-class correlation (ICC), and Lin’s concordance correlation coefficient (CCC). The primary criterion for model selection was out-of-sample log-likelihood.

The same steps were used for DCE, except for step 4, where out-of-sample log-likelihood was the only measure of performance.

All analyses were done in R for windows, and regression modelling was done using the *xreg* package [23].

## Results

The sociodemographic profile of the two samples is presented, against the most recent UK census data for comparability (Table 1).

For DCE, inclusion of an intercept substantially improved out-of-sample likelihood, while intercepts for cTTO reduced model performance. The best performing models in terms of all out-of-sample measures were hybrid variants with a DCE intercept and no cTTO intercept, all of which had better performance than corresponding models fitted to either data type in isolation. Out-of-sample accuracy statistics for the best performing models and the “base case” hybrid model can be found in Table 3. In- and out-of-sample accuracy statistics for all 16 tested models can be found in supplementary Table 1.

**Table 3:** Out-of-sample predictive accuracy for best performing models

	Log-likelihood			Pearson's R	ICC	CCC	MAE	RMSE
	TTO	DCE	Combined					
Hybrid models								
CALE	– 2755.2	– 4751.3	– 7506.4	0.9154	0.9012	0.9012	0.0672	0.0790
CALE separate for dimension groups	– 2756.4	– 4750.9	– 7507.3	0.9152	0.9017	0.9017	0.0633	0.0787
Base case hybrid	– 2776.6	– 4765.1	– 7541.7	0.8354	0.8280	0.8286	0.0734	0.0920
Separate DCE and cTTO models								
CALE	– 2784.2	– 5268.3	– 8052.5	0.8894	0.8693	0.8694	0.0761	0.0927
CALE separate for dimension groups	– 2788.3	– 5307.7	– 8096.1	0.8846	0.8632	0.8632	0.0784	0.0954
Base case hybrid	– 2778.9	– 4769.1	– 7548.0	0.8403	0.8372	0.8375	0.0729	0.0929

The chosen model, based on out-of-sample likelihood over cTTO and DCE data, was the hybrid CALE model. The model indicates substantial disutility associated with all eight dimensions of health, with the greatest losses associated with pain and depression and less difference between the other six dimensions. As level 6 was not included in either the cTTO or DCE survey, this has been interpolated as the mid-point between levels 5 and 7. The fitted coefficients, bootstrap-based standard errors, and predicted INQoL response level disutility values is shown in Table 4. The model indicates a comparatively large drop in utility between levels 5 (“a considerable amount”/”considerably”) and 7 (“an extreme amount”/”extremely”), and no additional utility loss between levels 3 (“a fair amount”) and 4 (“a moderate amount”/”moderately”).

**Table 4:**
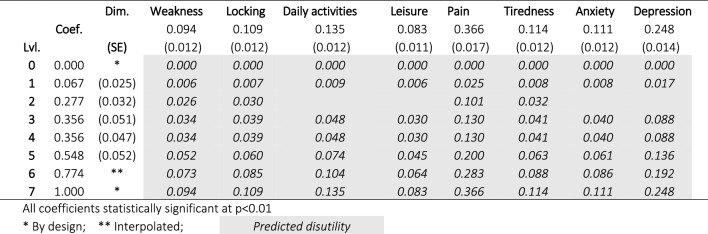
Coefficients and predicted disutility values for all level/dimension combinations from CALE hybrid

Table 5 summarizes the demographics of the two study samples, compared to the UK general population. The samples match the general population well in terms of gender, age, and ethnicity. We did find the number for female responders in the TTO study was higher than would be expected (58.5%) [24].

**Table 5:** Sample characteristics from valuation interviews

Characteristic	UK sample for DCE survey	UK sample for TTO valuation	UK sample DCE and TTO combined	UK population^a^
	*N* (%)			
*N*	508	200	708	
Age Mean (SD) Years	48.5 (16.3)	40.6 (13.6)	46.3	40.3
Sex				
Female	256 (50.4%)	117 (58.5%)	216.7 (52.7%)	50.6%
Ethnicity				
White	458 (90.2%)	153 (76.5%)	611 (86.3%)	84.8%
Asian (British, Indian, Pakistani, Bangladeshi) or Chinese	28 (5.5%)	22 (11%)	50 (7.1%)	7.5%
Black African, Caribbean, or Black British	4 (0.8%)	10 (5%)	14 (2.0%)	3.5%
Mixed race or multiple ethnicity	12 (2.4%)	11 (5.5%)	23 (3.2%)	1.8%
Other	6 (1.2%)	4 (2%)	10 (1.4%)	1.9%
Occupation				
Employed	268 (52.8)	143 (72%)	411 (58.1%)	60.5%
Retired	112 (22.1%)	21 (10%)	133 (18.7%)	–
In education or training	10 (2.0%)	20 (10%)	30 (4.2%)	–
Seeking work, unemployed	24 (4.7%)	7 (3.5%)	31 (4.4%)	2.5%
Homemaker/carer	44 (8.7%)	5 (2.5%)	49 (6.9%)	–
Other	50 (9.9%)	4 (2%)	54 (7.6%)	

^a^Figures based on data for 2019 United Kingdom (Office for National Statistics Population estimates for the UK, England and Wales, Scotland, and Northern Ireland – Office for National Statistics (ons.gov.uk); Population estimates by ethnic group and religion, England and Wales – Office for National Statistics (ons.gov.uk); Estimates for April to June 2019 employment status 16yrs + : Employment in the UK—Office for National Statistics (ons.gov.uk)). Please note the UK population mean age represents the total population, whereas the DCE survey and TTO exercise was restricted to adults

## Discussion

This report describes a study designed with advice from neuromuscular clinical experts and an expert in utility measurement to estimate utility weights from the INQoL – a quality of life measure developed specifically for use in people with neuromuscular conditions [5]. There are many difficulties in capturing reliable utility estimates in rare diseases because data are not always captured in clinical trials and stand-alone studies are difficult because of the low prevalence. In this study, we have used a combination of a sample of respondents valuing INQoL health using cTTO, boosted by a larger online sample comparing similar health states using DCE, to generate utilities in a hybrid model. This approach provides values on a suitable utility scale, informed by cTTO, while allowing for greater statistical power and improved representativeness through the use of a larger online sample. The tested hybrid models indicate improved out-of-sample predictive accuracy both for cTTO and DCE than corresponding models fitted to either data type in isolation. In other words, information from DCE responses improves our ability to predict cTTO responses, and vice versa.

There are a number of challenges with both surveys which we would like to discuss. Both methods rely on a restricted selection of INQoL items, based on a mix of capturing aspects central to myotonia, while retaining conceptual overlap with EQ-5D dimensions. Table 1 shows the conceptual relationship between the items, which we believe is quite substantial. Preferably such overlap should be statistically verified; however, we were unable to complete this step because we did not have access to a suitable dataset, which in this rare disease does not exist to the best of our knowledge.

Given the process leading to the selection of INQoL items for valuation, there is a substantial conceptual overlap with the EQ-5D, with some notable differences. Mobility (EQ-5D) is related to muscle weakness and muscle locking (INQoL), but it is not an exact match. Fatigue, which is not explicitly included in the EQ-5D, was included for valuation partly at the recommendation of the clinical experts but also because it is recognized to be an important feature of the disease. INQoL includes separate items for anxiety and depression whereas as EQ-5D has a single item covering both. Therefore, the scores do not necessarily reflect the level of both. However, given the level of conceptual overlap, we believe that the INQoL should align well with the descriptive system of the EQ-5D and therefore reflect generic elements of HRQoL as well as specific issues for people with NDM.

Commonly in economic evaluations of orphan drugs, decision makers are left to consider models which rely on expert opinion regarding outcomes, or the use of disease state vignettes. We have outlined some of the methodological limitations of our work for transparency. Despite the limitations we believe that the methods are an improvement over methods that are commonly used because they incorporate a validated patient HRQoL assessment.

These results demonstrate the potential advantages of hybrid modelling: by leveraging a combination of natively QALY-scaled preferences elicited using TTO with considerably less costly ordinal preferences elicited using DCE, we were able to achieve better coverage of the INQoL descriptive system, and a combined model that outperforms models separately fitted to each type of data. The mutually improved predictive accuracy supports the notion that TTO and DCE tap into the same underlying preference structure in INQoL valuation. In this study, the constrained CALE model approach resulted in better out-of-sample predictive accuracy than more flexible main-effects model alternatives. Applying cross-dimensional level constraints may be advantageous particularly when applied to large descriptive systems and in situations where the maximum feasible sample size is limited, e.g. due to time or costs.

There are important limitations with this work which should be considered when using the scoring algorithm. The DCE design was driven by the INQoL items and response options. The analyses identified some logical inconsistencies whereby specific logically worse responses were associated with point estimates indicating increasing utility. We applied some simple rules and assumptions so that the scoring weights did not include these inconsistencies. Emergence of these inconsistencies is not uncommon in such exercises [25], and in an online survey it is difficult to manage this, even when managed by an expert online provider. It was assumed that the subscribing respondents would likely have some experience in completing similar surveys of this kind. Nevertheless, the respondents were provided contact details to contact the facilitators to ask questions to support their understanding of the task at any time. Quality checks such as checking that no respondent always answered A or B were performed; however, some additional quality control checks may have identified if the task was not well understood. Additionally the DCE included eight attributes (for eight INQoL items) which in each choice task were all varied at the same time, which may have made the task too complex for some participants. Previous utility studies suggest that an individual can process between five and nine pieces of information at a time [25]; therefore, we acknowledge that the breadth of the descriptive system (8 dimensions) would be at the higher end of that range. The sample for the TTO study had a higher proportion of females than the UK population because of the use of convenience sampling. There is little evidence to suggest that preference weights for health states from women differ significantly from men (van Nooten et al. 2018) [24]. However, the over-representation of women means that the sample is not representative of UK society and so this remains a limitation. One other limitation arises from the selection of items. As described above, the INQoL items were selected because they were judged to have the most conceptual overlap with the items in the EQ-5D. So items on pain, depression and usual activities were selected for this reason. However, by selecting these items we may have missed important condition-specific items in the INQoL which contribute to its sensitivity as a disease-specific measure. This means that it is possible that the resulting preference-based scoring algorithm for the INQoL may not be as sensitive as the original measure. This could be an area for future research.

The valuation of the INQoL measure was based on two separate studies and the hybrid modelling provides a mechanism for combining these datasets. We believe that combining response data from both DCE and TTO may be particularly useful for capturing utilities in a rare disease such as NDM. As the hybrid approach provides values on a scale determined by responses to the TTO task, the INQoL utility values should be comparable to utility values from other TTO-based instruments, such as the EQ-5D. We would recommend the use of this INQoL value algorithm for use in QALY calculations in NDM where EQ-5D values do not exist. Indeed, it may be suitable for use in conditions beyond NDM, but this would require further empirical verification.

## Supplementary Information

Below is the link to the electronic supplementary material.

Supplementary file1 (DOCX 201 KB)

## Data Availability

The authors would be happy review requests for access to the study data which will be supported in most cases.
