# Supplementary material for: Preference-based utility weights for the Individualized Neuromuscular Quality of Life Questionnaire (INQoL), with a focus on non-dystrophic myotonia (NDM)
Source: Eur J Health Econ. 2024 Feb 28;25(8):1461–9. doi: 10.1007/s10198-024-01674-2 (PMC11639140; doi:10.1007/s10198-024-01674-2)
Supplement: Supplementary file 1 — Supplementary file1 (DOCX 201 KB) [file 10198_2024_1674_MOESM1_ESM.docx]

Appendix Figures & tables

Supplementary Figure 1: Example of a DCE choice set


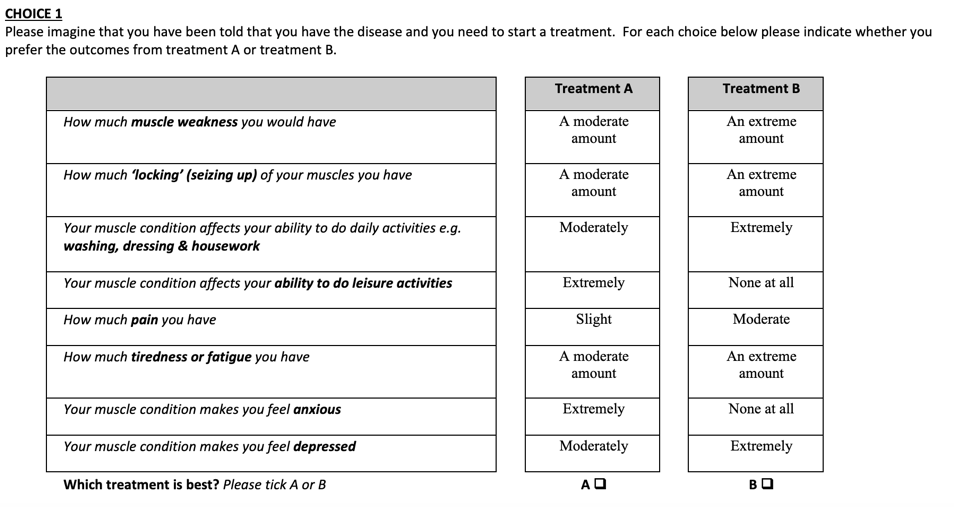


Supplementary Figure 2: Example of a Vignette used in the time trade off exercise


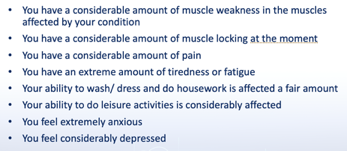


Supplementary Table 1: Complete in- and out-of-sample fit statistics
